# Mean value of B-mode optic nerve sheath diameter as an indicator of increased intracranial pressure: a systematic review and meta-analysis

**DOI:** 10.1186/s13089-021-00235-5

**Published:** 2021-07-02

**Authors:** Lisandro Montorfano, Qian Yu, Stephen J. Bordes, Shankarapryan Sivanushanthan, Raul J. Rosenthal, Miguel Montorfano

**Affiliations:** 1 Department of General Surgery, Cleveland Clinic Florida, 2950 Cleveland Clinic Blvd, Weston, FL USA; 2 School of Medicine, Georgetown University, Washington D.C, USA; 3 Department of Ultrasound and Vascular Doppler, Hospital de Emergencias “Dr. Clemente Alvarez”, Av. Pellegrini 3205, Rosario, Santa Fe, Argentina

**Keywords:** Trauma, traumatic brain injury, ocular ultrasound, Intracranial pressure, Optic nerve sheath, Optic nerve sheath diameter, Point-of-care ultrasound

## Abstract

**Objectives:**

Timely diagnosis and treatment of increased intracranial pressure can decrease morbidity and prevent mortality. The present meta-analysis aims to determine the mean value of the ONSD measured in patients with various elevated ICP etiologies under different clinical settings, as well as comparing the value of ONSD between patients with and without elevated ICP.

**Methods:**

This meta-analysis complied with the Preferred Reporting Items for Systematic Reviews and Meta-analysis Statement8. PubMed, Embase, and Cochrane Library were searched to identify ONSD measured by US for patients with increased ICP from establishment to October 2020.

**Results:**

A total of 779 patients with elevated ICP among 22 studies were included in the present meta-analysis. Studies were published between 2003 and 2020. Eighteen were comparative (18/22, 81.8%), and four were single-armed study (4/22, 18.2%). Twenty were prospective studies (20/22, 90.9%). There was moderate-to-high heterogeneity based on the prediction ellipse area and variance logit of sensitivity and specificity.

**Conclusions:**

The mean value of the ONSD among patients diagnosed with increased ICP was 5.82 mm (95% CI 5.58–6.06 mm). Variations were observed based on etiology of intracranial hypertension, clinical settings where ONSD was measured, and standards for diagnosing intracranial hypertension. The US-ONSD among patient with elevated ICP was significantly higher than the normal control. Although a cut-off value is not clearly determined, these mean values can be implemented to evaluate the sensitivity and specificity of US-ONSD in diagnosing intracranial hypertension in future studies.

## Introduction

Increased intracranial pressure (ICP) can arise from a variety of cerebral conditions such as stroke, bleeding, malignancy, and trauma [1]. Timely diagnosis and treatment of elevated ICP could prevent detrimental consequences such herniation and decrease mortality [2, 3]. Although invasive intracranial monitoring has been regarded the “gold-standard”, it demands neurosurgical expertise and is associated with post-procedural complications including infection, hemorrhage, and misplacement [4]. Signs of elevated ICP could manifest as effacement of ventricles and midline shift on CT and MRI [5], but these imaging modalities are not always available in all medical environment and require time for transfer. Within the last two decades, evidence has proven ultrasound (US) measurement of the optic nerve sheath diameter (ONSD) to be a surrogate for intracranial pressure, a point-of-care procedure that can be conveniently done at bedside [6, 7]. Increased ONSD has been noted in patients with elevated ICP of various etiologies in ICU, clinic, and emergency departments. Despite the currently available literature, there is no consensus regarding the cut-off of ONSD for elevated ICP. The present meta-analysis aims to determine the ONSD measured in patients with various elevated ICP etiologies under different clinical settings, as well as comparing the value of ONSD between patients with and without elevated ICP.

## Materials and methods

### Literature search

This meta-analysis complied with the Preferred Reporting Items for Systematic Reviews and Meta-analysis Statement [8]. PubMed, Embase, and Cochrane Library were searched to identify ONSD measured by US for patients with increased ICP from establishment to October 2020. The following keywords were used: “optic nerve sheath diameter”, “ultrasound”, “intracranial”, “pressure”, and “hypertension”.

### Inclusion criteria and exclusion criteria

The following inclusion criteria were adopted: (a) patients who underwent US measurement of ONSD; (b) patients with elevated ICP; (c) ONSD must be reported in mean and standard deviation on US; (d) confirmation of elevated ICP must be done in another modality other than ultrasound and clinical exam such as CT, MRI, lumbar puncture (LP), and invasive intracranial monitoring. A study was excluded if any of the following criteria were met: (a) non-clinical studies; (b) sample size less than 6; (c) case-reports; (d) pediatric and animal population, and (e) irrelevant studies that did not focus on the topic of using US to measure ONSD of patients with intracranial HTN.

Endnote X8 (Clarivate Analytics, Philadelphia, Pennsylvanian) was used to identify duplicates. Titles, abstracts, and key words were screened, followed by the review of full texts of the remaining studies.

### Data collection and statistical analysis

Baseline characteristics were extracted from each study including author, year of publication, country, study design, sample size, “gold-standard” of increased ICP, optic nerve diameter measured by US, hospital setting, etiology of increased ICP. “Gold-standard” is defined as increased ICP confirmed by CT, MRI, lumbar puncture (LP), or invasive intracranial monitoring. Optic nerve diameter was only included if reported in mean and standard deviation. Two researchers extracted the data from the original studies, and any disagreement was resolved through review and discussion.

Quantitative analysis was performed with Stata 15.1 (STATA Corp., College Station, TX, USA). Meta-analysis was conducted with the -*metan* function. Heterogeneity was assessed with the I^2^ statistics. A random-effect model was used for a more conservative estimation due to the quality of included studies. Optic nerve diameters were pooled if reported by original articles. Subgroup analysis was made based on hospital settings, etiology of increased ICP, and “gold-standard” of ICP measurement. Comparison of the increased ICP and normal ICP groups was performed via standard mean difference (SMD) by including comparative studies. Funnel plot and Egger’s tests were implemented to assess publication bias.

## Results

### Baseline characteristics

**Fig. 1:**
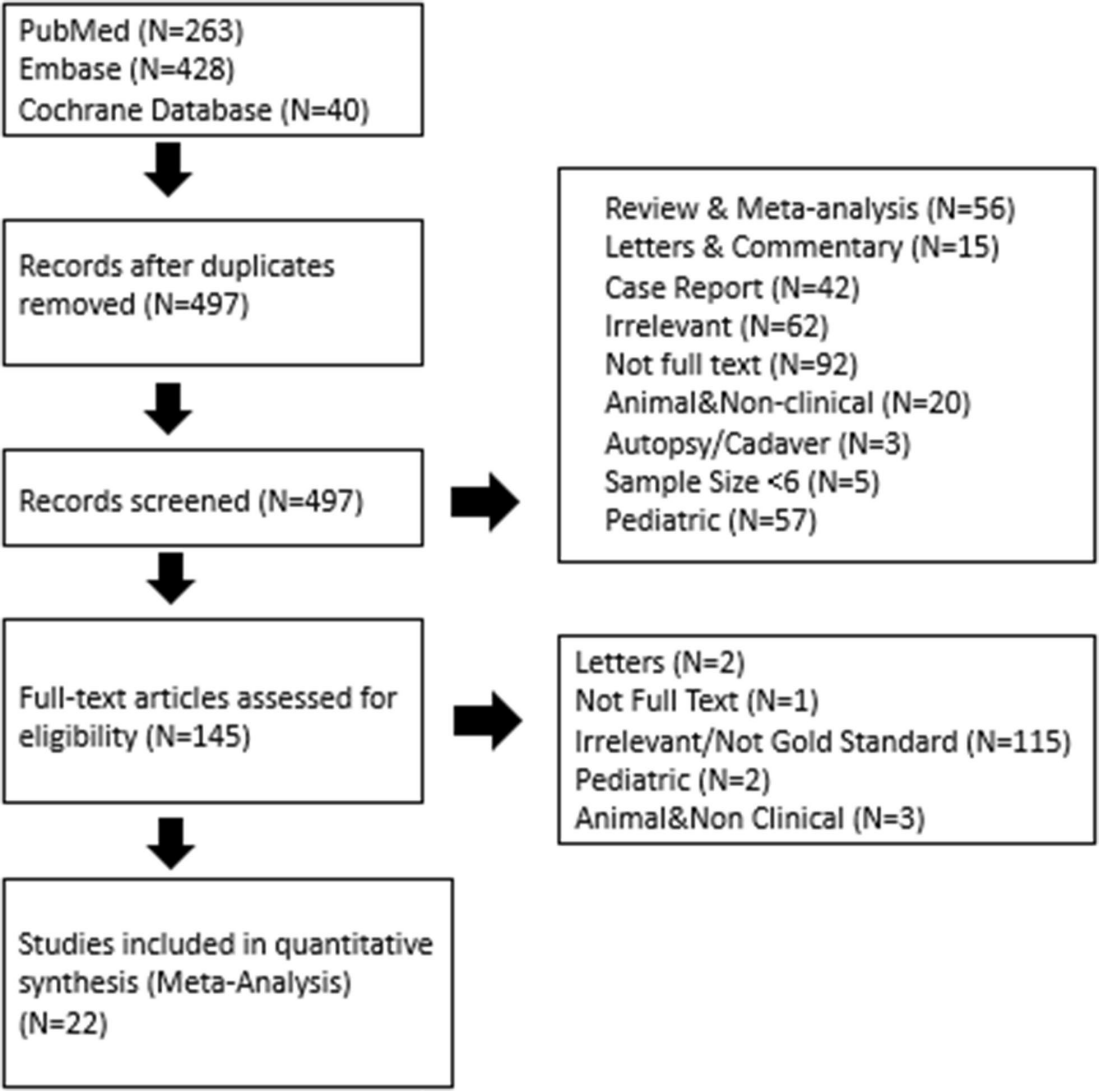
Flow-diagram of manuscript screening and selection

Among the initial 497 unique search results (Fig. 1), a total of 779 patients with elevated ICP among 22 studies were included in the present meta-analysis. Studies were published between 2003 and 2020. Eighteen were comparative (18/22, 81.8%), and four were single-armed study (4/22, 18.2%). Twenty were prospective studies (20/22, 90.9%). Studies were performed in USA (*n* = 3), China (*n* = 3), India (*n* = 3), Italy (*n* = 2), Turkey (*n* = 1), Thailand (*n* = 1), Spain (*n* = 1), Saudi Arabia (*n* = 1), Pakistan (*n* = 1), Nigeria (*n* = 1), Iraq (*n* = 1), Finland (*n* = 1), and France (*n* = 1). The brand of US machine implemented by each study is listed in Table 1. All studies measured ONSD at 3 mm posterior to the globe, with varying transverse and/or sagittal measurements and repetition times (Table 1).

**Table 1:** Baseline characteristics of included studies

Study/year	Region	Design	Sample size	Mean	Ultrasound brand	Clinical setting	Diagnosis	Etiology	Measurement technique
Aduayi 2015 [9]	Nigeria	Prospective	69	5.7	Mindray DC-6	Mixed	CT	Mixed	3 mm behind the globe; 3 times per eye; averaged
Altayar 2020 [10]	Saudi Arabia	Prospective	28	6.6	Unspecified	ICU	Inv	Trauma	3 mm posterior to the globe in transverse and sagittal planes in both eyes; average diameter was recorded
Bhadari 2019 [11]	India	Prospective	57	5.8	Unspecified	OR	Inv	Hydrocephalus	3 mm behind the posterior sclera
Blaivas 2003 [12]	USA	Prospective	14	6.27	Unspecified	ED	CT	Mixed	3 mm posterior to the globe for both eyes and then averaged.
Bäuerle 2011 [13]	Finland	Prospective	10	6.4	Philips iU22	Mixed	LP	IIH	3 mm behind the papilla. Measured 3 times and then averaged.
Chen 2019 [14]	China	Prospective	29	4.53	DelicaMVU-6300	Mixed	LP	Mixed	3 mm behind the globe; averaged two measurements: one sagittal and the other in the transverse plane
del Saz-Saucedo 2016 [15]	Spain	Prospective	19	6.76	Toshiba AplioXG	Mixed	LP	IIH	Transverse plane
Du 2020 [16]	China	Prospective	49	5.7	Sonosite	ICU	Inv	Trauma	3 mm behind the globe; transverse plane; averaged two eyes.
Geeraerts 2007 [17]	France	Prospective	15	6.3	HP Sonos 5500	ICU	Inv	Trauma	3 mm behind the globe; one sagittal and one transverse
Goel 2008 [18]	India	Prospective	73	5.8	Unspecified	Mixed	CT	Trauma	3 mm behind the globe; one sagittal and one transverse; averaged
Hanafi 2019 [19]	Iran	Prospective	62	6.06	SONOSCAPE-SSI 6000	ED	CT	Trauma	3 mm posterior to the globe; 3 times on each eye in an axial region; averaged.
Kimberly 2008 [20]	USA	Prospective	8	5.4	Sonosite Micromaxx	Mixed	Inv	Mixed	3 mm posterior to the orbit; axial image; six measurements; averaged.
Komut 2016 [21]	Turkey	Prospective	50	5.4	Toshiba Aplio 500 Platinum	ED	CT	Mixed	3 mm proximal to the optic disc; in transverse and sagittal planes.
Mohson 2019 [22]	Iraq	Prospective	40	5.6558	Philips HD11XE	Clinic	LP	Mixed	3 mm posterior to the globe; transverse.
Moretti 2009 [23]	Italy	Prospective	19	6.2	Hitachi EUB 405	ICU	Inv	Trauma	3 mm behind the globe; one sagittal and one transverse.
Nash 2015 [24]	USA	Prospective	9	5.8	SonoSite M-Turbo	Mixed	Inv	Trauma	3 mm posterior to the optic disk; longitudinal and transverse.
Rehman 2016 [25]	Pakistan	Cross-sectional	13	6.61	Toshiba Xario 200	Mixed	LP	IIH	3 mm behind the retina; 3 readings were averaged.
Shirodikar 2014 [26]	India	Prospective	35	5.43	Unspecified	Mixed	MRI	Mixed	3 mm behind the globe; averaging three readings from each eye.
Tarzamni 2016 [27]	Iran	Prospective	30	5.48	Aloka Model Prosound 3500	Mixed	CT	Mixed	3 mm posterior to the globe; transverse and sagittal; averaged.
Ussahgij 2020 [28]	Thailand	Prospective	29	5.3	SonoSite M-Turbo	ED	CT	Mixed	3 mm posterior.
Wang 2015 [29]	China	Cross-sectional	101	4.58	Philips iU22	Mixed	LP	Mixed	3 mm posterior to the orbit; transverse and sagittal; repeated twice; averaged
Zoerle 2020 [30]	Italy	Prospective	20	6.44	Phillips iE33	ICU	Inv	Trauma	3 mm behind the globe; transverse and sagittal; repeated twice; averaged

### ONSD measured by ultrasound

Based on 743 patients from 22 studies with elevated ICP, the overall pooled ONSD measured by ultrasound was 5.82 mm (95% CI 5.58–6.06). Based on etiology of increased ICP, patients who had trauma, hydrocephalus, and pseudotumor cerebri had ONSD of 6.12 (95% CI 5.88–6.35) mm, 5.80 (95% CI 5.64–5.96) mm, and 6.62 (95% CI 6.45–6.79) mm, respectively. By hospital settings, US ONSD that were measured in the ICU, ED, clinic, and OR had a diameter of 6.25 (95% CI 5.92–6.57) mm, 5.72 (95%CI 5.24–6.19) mm, 5.66 (95%CI 5.60–5.71) mm, and 5.80 (95% CI 5.64–5.96) mm, respectively. According to the diagnosis of increased ICP, patients who were diagnosed by CT, MRI, LP, and invasive intracranial monitoring had ONSD on US of 5.67 (95% CI 5.44–5.91) mm, 5.43 (95% CI 5.25–5.61) mm, 5.75 (95% CI 5.09–6.40) mm, and 6.04 (95% CI 5.76–6.32) mm, respectively.

### ONSD between patients with increased ICP and the normal cohort

Among 15 comparative studies, 463 and 609 patients had elevated and normal ICP, respectively. The ONSD measured by US was significantly larger among patients with elevated ICP compared to their normal counterparts (5.6 mm vs 4.1 mm, SMD: 2.44 [95% CI 1.93–2.95]). Similar findings were seen in each subgroup stratified based on gold-standard diagnosis modalities (CT, LP and invasive monitoring). Funnel plot did not show significant asymmetry (Egger’s test: p = 0.145).

## Discussion

The use of ultrasound in the hospital setting has increased drastically in recent years due to multiple factors such as advancements of technology, minimal invasiveness, affordability, ease of use, and increased access to providers [31–37]. Ultrasound is a valuable tool due to its ability to identify or rule-out certain pathologies quickly and efficiently [31–34]. As a result, it has been used in combination with gold standard techniques, which are oftentimes more invasive and require extended time.

The gold standard method to measure intracranial pressure (ICP) is intracranial monitoring with an intraventricular catheter, which typically requires an intraparenchymal catheter to be passed through a cranial burr hole into the ventricle. However, this test has multiple downsides even though it can determine the most accurate value [4, 38], including complications such as infection, post-procedural hemorrhage (due to device placement), parenchymal destruction, and misplacement in addition to other factors such as increased time for device placement and requirement for skilled in-house personnel, typically neurosurgeons [4]. Other less invasive tests that can identify increased ICP include radiological means such as CT and MRI, transcranial Doppler, ophthalmoscopy, tympanic displacement, and optic nerve sheath diameter [38]. As a result, these less invasive tests, especially those involving ultrasound, are frequently used as adjuncts or alternatives to more invasive monitoring. Recently, the use of optic nerve ultrasound as a noninvasive, accurate, safe, reproducible, and cost-effective tool for ICP via the measurement of optic nerve sheath diameter (ONSD) has been validated, thereby decreasing the potentially detrimental consequences of invasive transcranial measurements [6, 9–30].

**Fig. 2:**
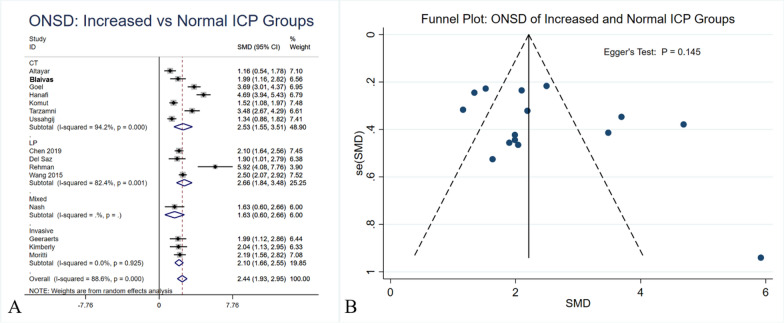
Optic nerve sheath diameter (ONSD) measured by ultrasound (US) between patients with elevated intracranial pressure (ICP) and normal cohort (**A**). Funnel plot and Egger’s test evaluating publication bias (**B**)

The anatomical relationship between the optic nerve and subarachnoid space results in an expansion of the optic nerve sheath in states of increased intracranial pressure [9, 12]. Thus, optic nerve sheath diameter can provide information regarding ICP. Ultrasound enables a noninvasive measurement to be taken 3 mm behind the globe, in a retro bulbar position, with a high frequency linear ultrasound probe, which allows for appropriate contrast between the optic nerve and retro bulbar fat to ensure accurate reference points [9, 12, 16]. Normally, three measures for each eye are taken and then averaged. Based on comparative studies, the US-ONSD among patients with elevated ICP was significantly greater than their normal ICP counterparts (Fig. 2A).

Nonetheless, the definitive mean value for ONSD in states of increased ICP has been a point of contention and controversy for many years due lack of meta-analysis and the inability to place an intracranial monitor in all patients where ICP is monitored to correlate this measurements in a more reliable way, though this value approximately lies between 5 and 6 mm based on prior literature [6, 9, 11, 16–18, 20–24, 26, 28]. In order to narrow this range to a more specific value, the present meta-analysis increases the power of the study by pooling available evidence in the literature. According to 22 unique studies over the past 17 years, the overall pooled ONSD measured by ultrasound was found to be 5.82 (95% CI 5.58–6.06) mm, consistent with previously reported MRI measurements (5.81–5.82 mm) [31, 32]. Subtle variations in mean values may be attributed to study site variation in diagnostic control (i.e., CT vs. MRI vs. LP vs. ICP monitor), patient variation such as anatomical differences or severity of illness (i.e., ICU status, etc.), and user variation such as ultrasound probe placement, reference points used for measurements, and the inter equipment variation related with ultrasound devices themselves (i.e., probe frequency, image quality, etc.). Widespread training in this practice is expected to increase precision and accuracy of measurements, and thus sensitivity and specificity of the exam findings.

**Fig. 3:**
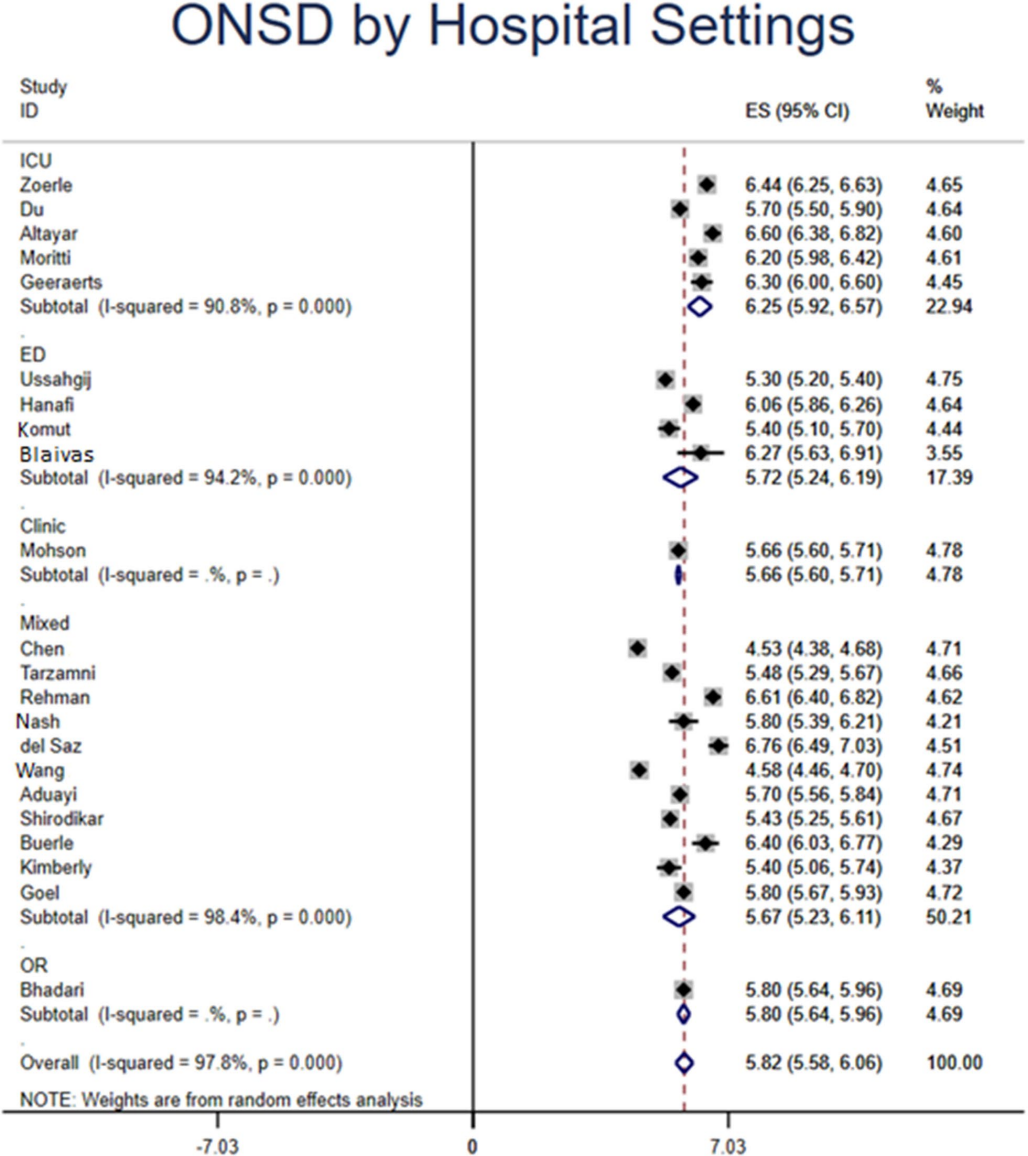
Optic nerve sheath diameter (ONSD) measured by ultrasound (US) stratified by clinical settings where measurement was taken place. *ICU* Intensive care unit, *ED* Emergency Department, *OR* operating room

Due to the easy accessibility of US compared to other imaging modality such as CT and MRI, it can be implemented by physicians in a variety of clinical settings, such as the ED, OR, ICU, clinic, wards, etc. (Table 1). US-ONSD measured in ICU patients demonstrated the largest value 6.25 (95% CI 5.92–6.57) mm (Fig. 3), reflecting the critically ill nature of this patient population and higher ICP requiring ICU care. Because the use of US is operator-dependent, the accuracy and precision of US-ONSD can be influenced by type of clinical setting where measurement takes place. For instance, measuring US-ONSD in a busy ED can be more technically challenging compared to in a controlled environment such as OR or ICU where patients are sedated. Further matched-comparative studies are warranted to compare its use in different clinical settings.

Previous studies indicated a strong correlation between increased ONSD and radiologic findings with CT and MRI [6, 9, 39, 40]. Patients diagnosed with increased ICP by CT or MRI had lower ONSDs on ultrasound (5.67 mm; 95% CI 5.44–5.91 mm and 5.43 mm; 95% CI 5.25–5.61 mm) than patients diagnosed with invasive ICP monitoring (6.04 mm; 95% CI 5.76–6.32 mm). Brain Trauma Foundation recommends considering invasive ICP monitoring under the setting of severe head injury [41]; the longer pooled ONSD of these patients in the present meta-analysis might be related to the severity of increased ICP prompted invasive monitoring.

**Fig. 4:**
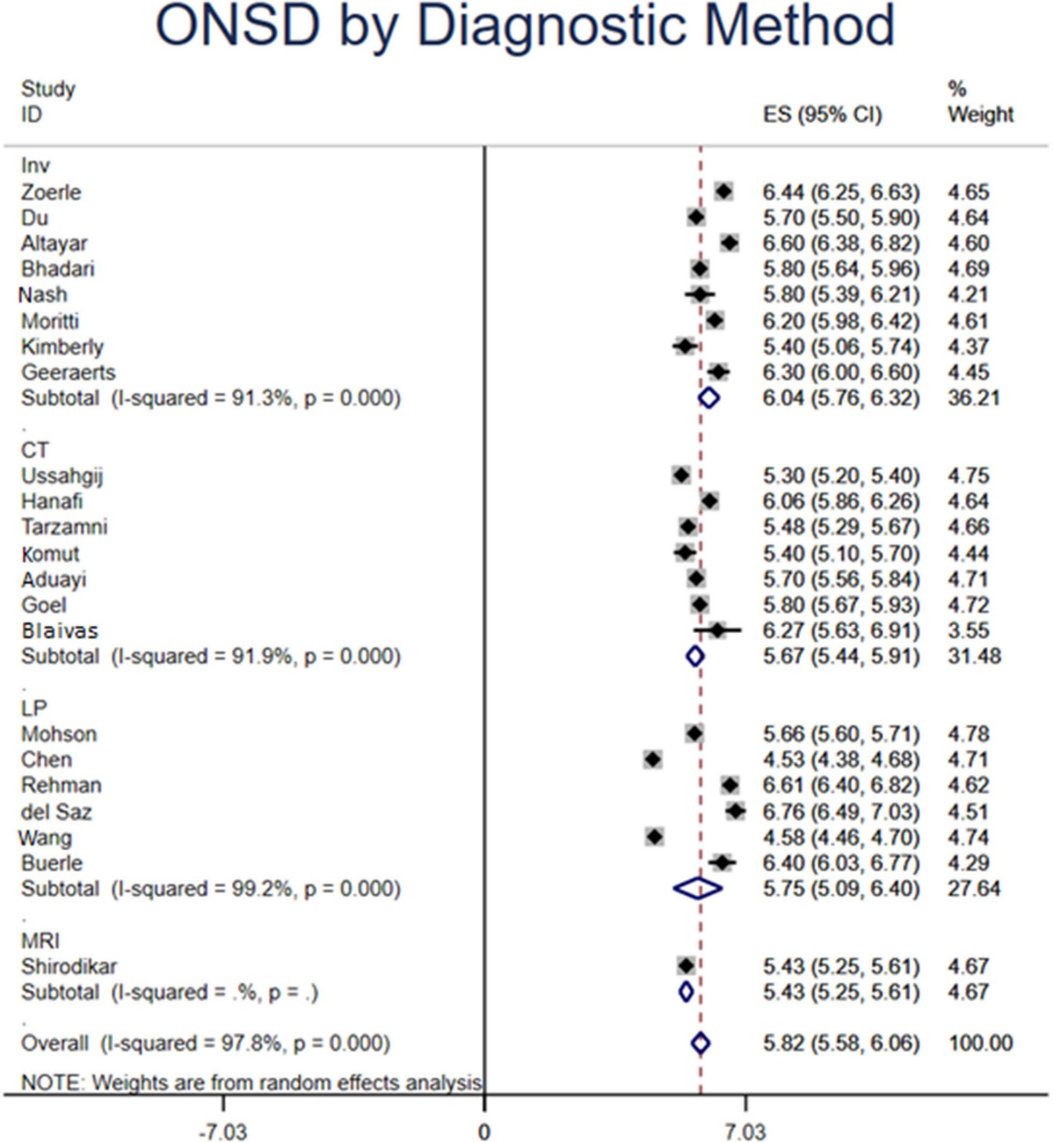
Optic nerve sheath diameter (ONSD) measured by ultrasound (US) stratified by the diagnostic method of increased intracranial pressure (ICP). *MRI* magnetic resonance imaging, *CT* computed tomography, *LP* lumbar puncture, *Inv* invasive intracranial monitoring

**Fig. 5:**
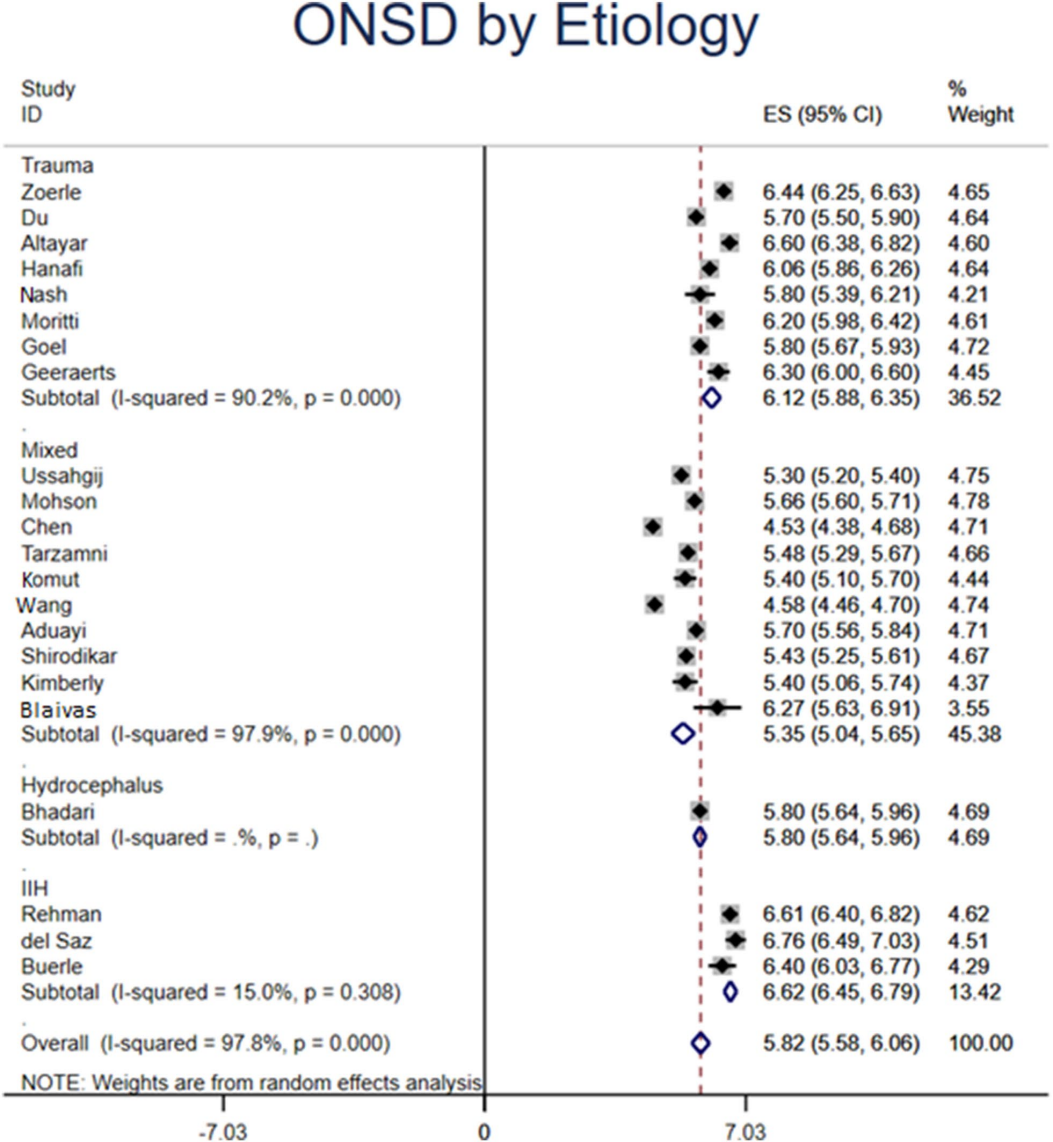
Optic nerve sheath diameter (ONSD) measured by ultrasound (US) stratified by etiology of elevated intracranial pressure

The present meta-analysis also found variations of ONSD among different etiologies of intracranial hypertension (Fig. 4), though there were only three study dedicated to IIT and one focusing on hydrocephalus. Compared to hydrocephalus, values for traumatic etiologies were greater than hydrocephalus (6.12 [95%CI 5.88–6.35] vs 5.8 [95%CI 5.64–5.96] mm), which may be explained by the rapid increase in ICP due to mass effect from head injury. However, the pooled pressure of idiopathic intracranial hypertension was the highest (6.62 [95%CI 6.45–6.79)] mm), which is possibly due to chronic change. Such observation could be affected by the etiology of the disease. Based on the modified Dandy criteria, papilledema is one criterion of diagnosing idiopathic intracranial hypertension [42]. Because papilledema is optic disc edema resulted from elevated ICP transmitted through optic nerve sheath [43], increase of ONSD should takes precedence prior to the development of papilledema. Because of under powering of the number of included studies, these speculation warrants further research (Fig. 5).

Fifteen studies compared the ONSD between patients with increased ICP and a normal cohort (Fig. 2). The ONSD measured by US was significantly larger among patients with elevated ICP compared to their normal counterparts (5.6 mm vs 4.1 mm, SMD: 2.44 [95% CI 1.93–2.95]).

The present study should be interpreted with several caveats. Despite most studies being prospective in nature, baseline patient characteristics can be heterogeneous such as the etiology of increased ICP and clinical settings where US-ONSD measurement was taken place. Subgroup analyses were performed and showed variations in terms of the pooled value in each condition. Nonetheless, the majority of studies were reported ONSD among a mixed population. Future study should report outcomes stratified based on these variables to determine the mean value for elevated ICP in each clinical scenario. Further, the present meta-analysis did not analyze the sensitivity and specificity of the pooled US-ONSD value. Such analysis requires each individual study reporting sensitivity and specificity in the original article, but the criteria of US-ONSD for elevated ICP vary among different authors. A number of studies for the ONSD evaluation used the median values rather than the mean value. Although in our analysis we used all the values relevant in the literature to obtained mean values, this could be a potential confounder when trying to establish an accurate value. ONSD values are usually represented as the average of measurements between the eyes. Normally, three measures for each eye are taken and then averaged. However, some studies used only two measures per eye, and some others used one transverse measurement and one on vertical plane per eye (averaged). Furthermore, some studies have suggested an "internal" ONSD (ONSDi) and an "external" ONSD (ONSDe). The two measurements can differ. These factors can produce differences of measurements and potential bias may be created.

Even if there are several limitations about the use of a unique cut-off value of ONSD for detecting elevated ICP, this update may help to clarify which is the value that could be considered as marker of intracranial hypertension.

Thus, the aim of the present study was to pool the US-ONSD using available studies to determine a mean value that can be used as a reference for future study to determine its specificity and sensitivity in real-world clinical practice.

## Conclusions

Based on available evidence, the mean value of the ONSD among patients diagnosed with increased ICP was 5.82 mm (95% CI 5.58–6.06 mm). Variations were observed based on etiology of intracranial hypertension, clinical settings where ONSD was measured, and standards for diagnosing intracranial hypertension. The US-ONSD among patients with elevated ICP was significantly higher than the normal control. Although a cut-off value is not clearly determined these mean values can be implemented to evaluate the sensitivity and specificity of US-ONSD in diagnosing intracranial hypertension in future studies. Larger, stratified, prospective studies are warranted to determine its value in each clinical setting.

## Data Availability

All data are available from the authors upon request.
